# Psychological Distress and Coping Mechanisms Among Flood-Affected Children in Maiduguri, Nigeria

**DOI:** 10.3390/children12091137

**Published:** 2025-08-28

**Authors:** Habu Haruna, Robert Kever, Babaji Maigari, Inuwa Ahmadu, Dathini Hamina, Dauda Salihu, Umar N. Jibril, Muhammad Chutiyami

**Affiliations:** 1 Department of Nursing Sciences, Faculty of Allied Health Science, College of Medical Sciences, University of Maiduguri, Maiduguri 600104, Nigeria; habaharun@unimaid.edu.ng (H.H.); robertkever72@unimaid.edu.ng (R.K.); babajimaigari@unimaid.edu.ng (B.M.); ahmaduinuwa@unimaid.edu.ng (I.A.); dathinihamina@unimaid.edu.ng (D.H.); 2 Department of Psychiatry and Mental Health Nursing, College of Nursing, Jouf University, Sakaka 72388, Saudi Arabia; 3 Department of Nursing Sciences, University of Ilorin, Ilorin 240003, Nigeria; umar.nj@unilorin.edu.ng; 4 School of Nursing and Midwifery, Faculty of Health, University of Technology Sydney, Sydney 2007, Australia

**Keywords:** psychological distress, coping mechanisms, flood disaster, children

## Abstract

**Background**: Flood disasters, alongside prolonged conflict and socioeconomic hardship in Maiduguri, Borno State, Nigeria, have heightened the psychological vulnerability of children. This study examined the prevalence of psychological distress and explored the coping mechanisms employed by children affected by flooding in the region. **Method**: Children aged 7–17 years from flood-affected areas in Maiduguri were included in the study. Psychological distress was measured using the parent version of the Strengths and Difficulties Questionnaire (SDQ-13), and coping mechanisms were assessed using the KidCOPE parent version. Multivariate and ordinal logistic regression examined factors associated with psychological distress and coping mechanisms. **Results**: A total of 374 children participated in the study. A total of 63.6% experienced abnormal psychological distress. Moderate and high levels of maladaptive coping were significantly associated with greater odds of psychological distress (odds ratio [OR] = 1.72, 95% CI: 1.25–2.36; OR = 2.43, 95% CI: 1.46–4.04). Similarly, moderate adaptive coping was associated with higher odds of distress compared to poor coping (OR = 1.90, 95% CI: 1.38–2.61). In unadjusted models, age, female gender, higher education, Christian religion, and higher household income were associated with increased psychological distress. However, these were not significant in the adjusted model. Ordinal logistic regression showed no significant predictors of either maladaptive or adaptive coping levels. **Conclusions**: A high proportion of flood-affected children in Maiduguri experience psychological distress, with maladaptive coping playing a key role. The findings indicate the need for targeted psychosocial interventions to improve adaptive coping skills in flood-affected children.

## 1. Introduction

Floods rank among the most devastating natural disasters worldwide, causing widespread destruction, displacement, and significant psychological trauma, especially among vulnerable populations like children. Children are particularly susceptible due to their developmental stage and dependence on caregivers for emotional stability and protection [1]. The psychological distress experienced by children affected by floods often manifests as anxiety, depression, post-traumatic stress disorder (PTSD), and emotional withdrawal [2]. These outcomes are often exacerbated by the loss of loved ones, damage to homes, and disruption of schooling and social support systems, all of which can hinder children’s emotional, cognitive, and social development [3].

In Nigeria, flooding has become increasingly frequent due to climate change, poor urban planning, and inadequate drainage infrastructure [4]. Flooding is also predicted to be a major problem in Northeastern Nigeria—Borno, Yobe, and Adamawa states [5], which could exacerbate the existing cases of child marriage, malnutrition, and disease [2]. Maiduguri, the capital of Borno State, is particularly vulnerable not only to seasonal flooding but also to the prolonged effects of insurgency and displacement [6]. This dual burden of climate-related disaster and armed conflict has placed enormous stress on already fragile communities, especially children, who are exposed to both environmental and sociopolitical traumas [6]. Armed conflict and displacement have become a persistent backdrop since the start of the Boko Haram crisis over a decade ago, arguably forming a new baseline of adversity for many children in the region [6]. The seasonal flooding, coupled with a breach in Alau Dam (a major state water reservoir), which submerged half of the entire city of Maiduguri [2], was an extraordinary and acute event. This led to catastrophic flooding, widespread displacement, and the destruction of homes, schools, and livelihoods, significantly intensifying the community’s existing vulnerabilities. Such compounded stressors elevate the risk of psychological distress and can contribute to long-term developmental and mental health challenges [7].

Although the mental health impacts of flooding are globally acknowledged, there remains limited research specifically focused on the psychological well-being of children in Maiduguri. Most interventions in the aftermath of floods have emphasized physical relief efforts, such as food distribution, shelter, and sanitation, while psychosocial support services remain limited or absent [1,8]. Existing literature identifies symptoms such as fear, depression, social withdrawal, and concentration difficulties in children post-disaster, which can persist for extended periods and negatively affect academic performance, peer interactions, and overall well-being [9].

Coping strategies play a crucial role in determining how children adapt to traumatic events. Adaptive coping mechanisms, such as seeking social support, engaging in play therapy, or reframing traumatic experiences, can foster resilience and recovery. In contrast, maladaptive coping mechanisms, including avoidance, aggression, and emotional detachment, can exacerbate psychological distress [7,9]. The specific coping methods children employ are influenced by various factors, including their developmental stage, family environment, access to mental health resources, and community support systems.

Despite extensive research on coping mechanisms in various disaster-prone contexts, there remains a significant gap in understanding how children in Maiduguri, an area affected by both recurrent flooding and prolonged insurgency, navigate psychological distress. While studies such as Masten and Narayan [9] have outlined the pathways of risk and resilience in children exposed to disasters, these works primarily reflect global or generalized contexts and do not account for the unique interplay of conflict and environmental disasters in North-East Nigeria. In Maiduguri, the dual burden of armed insurgency and climate-induced flooding has created an especially vulnerable population of children, yet most post-disaster responses have focused on physical relief efforts like food and shelter [1], with little attention to mental health and psychosocial support. Therefore, this study seeks to assess the prevalence of psychological distress among flood-affected children in Maiduguri, Nigeria, and to explore the coping mechanisms they employ in response to these layered adversities. Based on the reviewed literature and contextual factors, the following hypotheses were set to guide the conduct of this study:

(1) Children who demonstrate higher levels of maladaptive coping will have significantly greater odds of experiencing abnormal psychological distress compared to those with lower levels of maladaptive coping.
(2) Sociodemographic variables such as age, gender, educational status, and household income will be significantly associated with levels of psychological distress and coping mechanisms. These insights will contribute to the development of evidence-based, child-centered mental health responses for disaster-affected populations in conflict-impacted regions.

## 2. Methodology

A descriptive cross-sectional research design was adopted for this study. This is because the design gives a snapshot of a situation at a specific point in time by collecting data from a group of individuals to describe the characteristics or prevalence of a particular condition or phenomenon within that population [10,11]. Therefore, a descriptive cross-sectional research design was used to assess psychological distress and coping strategies among flood-affected children in Maiduguri, Nigeria, thereby capturing the prevalence of distress and related coping responses [12,13]. The study was conducted in Maiduguri, the capital of Borno State, Nigeria. This region, particularly Maiduguri, has been repeatedly affected by seasonal flooding, which has compounded the vulnerabilities already caused by over a decade of armed conflict and internal displacement.

**Figure 1:**
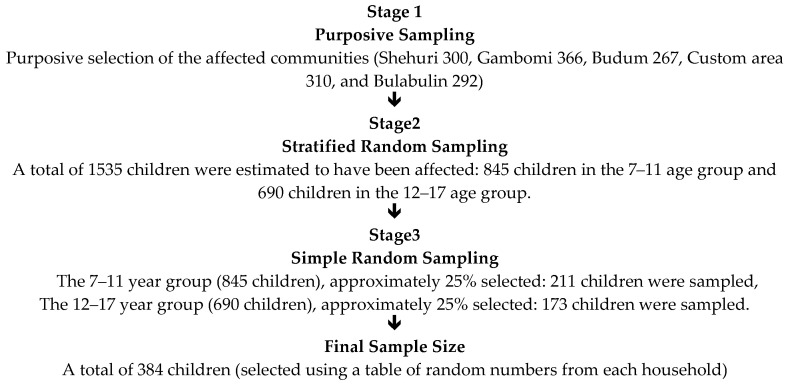
FLOW CHART indicating sampling process.

A multi-stage sampling technique was employed for this study to ensure the representativeness of the flood-affected children in Maiduguri (Figure 1). This was necessary to ensure the affected population’s diverse demographic and geographic characteristics are captured. To achieve this, the sampling process involved three main stages. First, purposive sampling was used to select specific communities affected by the flood in Maiduguri, Borno State, Nigeria. These communities were identified with the support of local health authorities, community leaders, and humanitarian actors. This intentional selection focused on areas where children had experienced direct flood-related trauma and displacement.

Subsequently, the selected flood-affected areas were stratified based on age groups, specifically children aged 7–11 years and those aged 12–17 years. This stratified random sampling approach was used because psychological distress and coping mechanisms may vary significantly between younger children and adolescents, as age has been shown to significantly influence children’s emotional responses to disasters and the coping mechanisms they adopt [14]. Finally, within each age stratum, a simple random sampling technique was used to select participants, represented by their parents or primary caregivers. A table of random numbers was employed to ensure unbiased selection within each household list compiled in the affected communities. This method ensured that every eligible child had an equal chance of inclusion, thereby reducing selection bias.

The sample size for this study was determined using Cochran’s formula for cross-sectional studies. Therefore, the formula *n* = Z^2^ P (1 − P)/d^2^ [15] was used, where *n* is the sample size, Z is the Z-score corresponding to the desired confidence level (1.96 for a 95% confidence level), P is the estimated proportion of the population (set at 0.5 or 50% in the absence of prior studies in the study area), and d is the margin of error (set at 0.05, or 5%). The estimated prevalence of psychological distress among flood-affected children was 0.5 (50%) due to the absence of prior studies in the study area. Therefore, the sample size was 384 parents/caregivers of the affected children in the flood-affected areas using the aforementioned formula.

Regarding the inclusion and exclusion criteria for this study, only children aged 7–17 years who had experienced flooding in Maiduguri, lived in affected areas for at least six months, and whose parents/guardians provided consent to be part of the study. Children with severe cognitive or developmental disabilities or a lack of consent were excluded.

The Strengths and Difficulties Questionnaire (SDQ-13) parent version (https://www.sdqinfo.org/ accessed on 21 July 2025) was adopted to assess psychological distress. The SDQ-13 parent version is a brief behavioral screening tool designed to assess emotional and behavioral difficulties in children and adolescents based on parental reporting. Participants’ distress levels were categorized based on their SDQ-13 scores, which range from 0 to 26, with higher scores indicating greater distress. Using the SDQ scoring system, we categorized scores between 0 and 13 as “normal” distress levels, indicating no significant emotional or behavioral difficulties, while scores between 14 and 26 were classified as “abnormal” distress levels, indicating clinically significant emotional and behavioral difficulties. This categorization was based on established cutoff scores for the SDQ-13, which reflect levels of distress that are clinically significant and warrant attention, especially in populations exposed to severe stressors, such as those in Maiduguri. For the coping strategies, a 15-item parent version of KidCOPE (https://pedpsych.org/wp-content/uploads/2022/09/Kidcope-KidCOPE.docx, accessed on 21 July 2025) was adopted to determine the coping strategies. The 15-item parent version of KidCOPE is an adapted form of the original child self-report questionnaire designed to assess parental perceptions of the coping strategies their children use in response to stress or trauma. In this study, for Adaptive Coping (items 1–6 and 15), with a range of 0–14, participants scoring 0–4 indicate Poor Adaptive Coping, 5–9 Moderate Adaptive Coping, and 10–14 Strong Adaptive Coping. Also, for Maladaptive Coping (items 7–14) with a range of 0–16, participants scoring 0–5 indicate Poor Maladaptive Coping, scores of 6–10 Moderate Maladaptive Coping, and participants scoring 11–16 demonstrated High Maladaptive Coping. Both instruments (SDQ-13 and KidCOPE) have been validated and used in similar low-income and humanitarian settings in sub-Saharan African contexts [14,16].

Data were collected through face-to-face, interviewer-administered questionnaires conducted by trained research assistants using English, Hausa, or Kanuri based on participant preference. This was due to low literacy levels in the study area. To maintain participant confidentiality and encourage candid disclosure, interviews were held in quiet, polite settings within communities or camps for displaced people. The Federal Ministry of Health Research Ethics Committee, through the Borno State Ministry of Health Research Ethics Committee, granted ethical approval for the study (NHREC/082/12/2024). All participating children’s parents or legal guardians provided written informed consent. The confidentiality of their answers was guaranteed, and participants were made aware of their freedom to leave the study at any time without facing any consequences. According to the Declaration of Helsinki [17], the values of beneficence, justice, and respect for persons were rigorously upheld during the entire research process.

All statistical analyses were conducted using IBM SPSS Statistics for Windows, version 30.0.0 (IBM Corp., Armonk, NY, USA). Descriptive statistics, including frequencies, percentages, means, and standard deviations, were computed to summarize sociodemographic characteristics and levels of psychological distress and coping mechanisms among participants. To examine the relationships between psychological distress and levels of coping mechanisms (maladaptive and adaptive), binary logistic regression analysis was performed. Odds ratios (ORs) with 95% confidence intervals (CIs) were calculated, and statistical significance was determined at *p* < 0.05. Both univariate (crude) and multivariate (adjusted) logistic regression models were estimated to identify sociodemographic factors associated with psychological distress. Further, to explore predictors of coping strategies (adaptive and maladaptive), an ordinal logistic regression analysis was conducted. Coping levels were treated as ordinal dependent variables (poor, moderate, strong for adaptive; low, moderate, high for maladaptive). Estimates and 95% confidence intervals were reported. Statistical significance was considered at *p* < 0.05. Appropriate model assumptions, including scales of measurements and multicollinearity, were adhered to, and missing data were excluded listwise.

## 3. Results

This study assessed psychological distress and coping mechanisms employed by flood-affected children in Maiduguri, Nigeria. A total of 384 questionnaires were distributed, but only 374 met the criteria for inclusion in the analysis. Therefore, the return rate was 97.4%.

Table 1 presents the sociodemographic characteristics of the respondents, along with the distribution of key study variables, including psychological distress, maladaptive coping, and adaptive coping. The sample consisted of 374 participants, with a slightly higher proportion of males (56.1%) compared to females. Most children were not enrolled in formal education (41.2%), and over half of the fathers (54.3%) had no formal education, with only 6.1% having attained tertiary education. In terms of religion, a majority of respondents identified as Muslim (63.1%), and most households earned between 21,000 and 30,000 (Naira) local currency per month (41.4%). The mean age of the children was 11.2 years.

Regarding the outcome variables, 63.6% of children were classified as having abnormal psychological distress, while 36.4% were classified as normal. For coping styles, maladaptive coping was prevalent, with 43.6% reporting moderate levels and 19.3% high levels. In contrast, adaptive coping was generally poor, with over half (53.8%) of the children showing poor adaptive coping, and only 2.0% exhibiting strong adaptive coping. Additionally, the distribution of psychological distress by levels of coping is presented in Supplemental Figures S1 and S2.

In Table 2, the regression analysis showed that higher levels of maladaptive coping were associated with increased odds of psychological distress. Compared to those with low maladaptive coping, children with moderate and high maladaptive coping had significantly greater odds of experiencing psychological distress. Similarly, those with moderate adaptive coping had higher odds of distress compared to those with poor adaptive coping. However, strong adaptive coping did not show a significant association, likely due to the small sample size in that category.

The binary logistic regression (estimated as the crude odds ratio) analysis identified several socioeconomic factors significantly associated with abnormal psychological distress. In the unadjusted models, older age, female gender, father’s primary school education, Christian religion, and both high and low household income were significantly associated with increased odds of psychological distress. However, in the multivariate model (estimated as adjusted odds ratio), no predictors remained statistically significant, suggesting potential confounding effects. The direction of associations remained generally consistent (Table 3).

Table 4 indicates ordinal logistic regression analysis results, which did not reveal any statistically significant predictors of adaptive coping. While age, male gender, and higher education levels showed some positive associations with higher adaptive coping, none reached statistical significance. Confidence intervals for all predictors included zero, suggesting a lack of strong association.

Similarly, no sociodemographic variables were found to be statistically significant predictors of maladaptive coping. Estimates for age, gender, education, religion, and economic status showed no significant associations with coping levels, and all confidence intervals crossed zero.

## 4. Discussion

This study assessed flood-affected children’s psychological distress and coping mechanisms in Maiduguri, Nigeria. The findings of the study revealed that 63.6% of children exhibited abnormal psychological distress, while 36.4% were classified as normal. For coping strategies, maladaptive coping was prevalent, with 43.6% reporting moderate levels and 19.3% high levels. In contrast, adaptive coping was generally poor, with over half (53.8%) of the children showing poor adaptive coping, and only 2.0% exhibiting strong adaptive coping. These findings align with the author of [18], who reported that 48.0% of the children in their study were found to have emotional and behavioral problems (EBP)**.** This indicates that nearly half of the children displayed signs of psychological or behavioral difficulties, such as emotional distress, conduct issues, or problems with social interaction. In light of recent discussion on climate anxiety and ecological emotions, our findings add to growing global concern over how children and adolescents emotionally respond to environmental disasters. Hickman et al. [19], in one of the largest global studies on climate anxiety, reported that over 75% of young people from countries such as Nigeria felt the future was frightening due to climate change, with many experiencing sadness, anxiety, and helplessness. This aligns with our study’s findings of high psychological distress among flood-affected children, suggesting that environmental disasters such as flooding intensify climate-related emotional responses, even in conflict-affected regions.

The findings of the study revealed that 63.6% of the flood-affected children showed abnormal psychological distress, which suggests high emotional and behavioral vulnerability. This finding may not be unconnected to the multifaceted trauma experienced by children in the study area, not just from natural disasters like flooding but also from unending conflict, displacement, and poverty. In agreement with this finding is a study by David et al. [20] and Cénat et al. [21], who reported significant symptoms of psychological distress among displaced adolescents in Northeast Nigeria.

The findings of this study revealed that 53.8% of respondents had poor adaptive coping. This implies that a significant number of the children in the study area lack effective psychological strategies to cope with the post-flood effects. Adaptive coping mechanisms are known to serve as critical shields against psychological distress, especially in the context of trauma. Given that 41.2% of children in this study do not have or are not enrolled in any formal education, it is possible that these children had fewer opportunities to learn and practice adaptive coping. This highlights the interconnectedness of education, psychosocial well-being, and disaster. This finding aligns with Ghasemi et al. [22], who reported that under conditions of extreme stress, children with access to protective interpersonal relationships and internal coping strategies are more resilient and less likely to experience long-term emotional problems. These findings also align with Masiran et al. [18], who reported that 48.0% of the children in their study were found to have emotional and behavioral problems (EBP). This indicates that nearly half of the children displayed signs of psychological or behavioral difficulties, such as emotional distress, conduct issues, or problems with social interaction.

However, in regions like Maiduguri, the development of such protective mechanisms is hindered by repeated exposure to conflict, displacement, poverty, and natural disasters. It is important to state that children who have been uprooted from familiar environments, exposed to repeated losses, or denied safe spaces and education are at a higher risk of developing maladaptive coping patterns due to chronic stress and emotional insecurity [23]. In a study conducted among displaced adolescents in Northeast Nigeria, David et al. [20] found that many exhibited signs of psychological distress, including sadness, isolation, and hopelessness, largely due to a lack of supportive environments and mental health services. Similarly, the present study shows that 43.6% of children reported moderate maladaptive coping, while 19.3% demonstrated high levels of such behavior. Maladaptive coping, such as avoidance, emotional withdrawal, aggression, or denial, may serve as short-term relief mechanisms but are typically associated with poor psychological outcomes when persistent. David et al. [20] and Kar [13] noted that children who lacked coping resources often developed social withdrawal and engaged in risky behaviors as a means of dealing with overwhelming stress. Additionally, Sheikh et al. [24] observed that trauma-exposed internally displaced children in Kaduna, Nigeria, exhibited patterns of emotional numbing and behavioral disturbances, especially when psychosocial interventions were not provided.

The regression analysis showed that higher levels of maladaptive coping were strongly associated with increased odds of psychological distress. Compared to those with low maladaptive coping, children with moderate and high maladaptive coping had significantly greater odds of experiencing psychological distress. Similarly, those with moderate adaptive coping had higher odds of distress compared to those with poor adaptive coping. However, strong adaptive coping did not show a significant association, likely due to the small sample size in that category. The unexpected association between moderate adaptive coping and higher distress may reflect a transitional or mixed coping phase in which children attempt to use some adaptive strategies, but in the face of overwhelming and compounding stressors (e.g., flooding, conflict, displacement), these strategies may be insufficient or inconsistently applied. Alternatively, it is possible that children with greater psychological distress are more likely to attempt adaptive strategies, but these efforts are not yet effective enough to reduce their distress, particularly in the absence of supportive environments or professional guidance. The findings align with a meta-analysis by Raccanello et al. [25] found that maladaptive coping strategies like escape and social isolation significantly correlate with higher levels of post-traumatic stress disorder (PTSD), depression, and anxiety among children and adolescents following natural disasters. Also, a study by David et al. [20] focusing on internally displaced adolescents in Northeast Nigeria reported that reliance on maladaptive coping mechanisms, including social withdrawal and risky behaviors, exacerbated psychological symptoms and hindered recovery. Our findings also resonate with international studies on children’s emotional reactions to floods in other contexts. For example, Pfefferbaum et al. [26] and Majumder et al. [27] demonstrated elevated symptoms of mental health disorders such as anxiety and PTSD among children exposed to floods and other extreme weather events in the US and Southeast Asia, respectively. While these contexts differ socioeconomically from Maiduguri, they collectively highlight that flood disasters universally disrupt children’s mental well-being. However, the compounded stressors of armed conflict, displacement, and poverty in Nigeria arguably create a more complex psychological burden. This underlines the need to strengthen research and intervention strategies tailored to the realities of the Global South.

The findings from this study reveal a complex relationship between sociodemographic factors and psychological distress among children affected by flooding. Initially, age was significantly associated with psychological distress in the unadjusted model (OR = 1.05; 95% CI: 1.03–1.06; *p* < 0.001), suggesting that older children experience greater emotional difficulties. However, this association diminished in the adjusted model (OR = 1.00; 95% CI: 0.96–1.04), indicating that the relationship may be confounded by other variables such as socioeconomic status or education. This pattern aligns with evidence from sub-Saharan Africa, where older children exhibit heightened emotional vulnerability due to greater cognitive awareness of traumatic events [28]. Furthermore, older children may assume caregiving roles or be more directly exposed to the consequences of displacement, which can exacerbate psychological burden.

Gender differences in distress were also observed. Female children were more likely to experience psychological distress in the crude analysis (OR = 1.65; 95% CI: 1.20–2.26; *p* < 0.01), though the association was not statistically significant in the adjusted model. This finding mirrors other studies indicating that girls often report higher levels of psychological distress, particularly in humanitarian settings. Such disparities are frequently attributed to cultural expectations, gender-based violence, and psychosocial vulnerabilities [28]. Girls are often socialized to internalize stress, making them more prone to anxiety and depression in the aftermath of crises. Educational status was initially related to increased psychological distress, especially among children in primary and secondary school. This may arise from educational interruptions, ambiguity regarding future education, and increased performance expectations. This finding is in agreement with Tol et al. [29], who assessed mental health and psychosocial support in humanitarian settings. Paternal education initially showed a strong association with child distress (OR = 2.42; 95% CI: 1.62–3.64; *p* < 0.001), but this effect also diminished in the adjusted model. This might reflect the psychological pressure placed on children by parents with educational backgrounds who expect better outcomes despite the challenges posed by displacement. While parental education is generally linked to favorable results, in emergency contexts it may serve as a source of stress when objectives become unachievable [30].

Religious affiliation appeared to be a significant factor in the unadjusted analysis, with Christian children reporting higher distress levels than their Muslim peers. However, this association became non-significant after adjusting for confounders. Religious minority status may contribute to heightened psychological vulnerability, particularly when it involves reduced access to communal support or heightened social isolation in regions dominated by a different faith group [31]. Interestingly, children from middle- and high-income households initially reported higher psychological distress. This counterintuitive trend may result from a steep decline in living standards, leading to greater psychological disruption in families unaccustomed to hardship. Economic shocks can affect identity, stability, and coping resources [32].

Overall, the findings of the current study suggested several sociodemographic factors were linked to psychological distress; these associations weakened after adjusting for confounders. This highlights the complexity of children’s psychological responses and the influence of cumulative stressors on individual characteristics. Importantly, ordinal regression analysis revealed that no sociodemographic variable significantly predicted adaptive or maladaptive coping. These results are consistent with Batte et al. [33], who found that in disaster contexts, the severity of shared stressors can minimize typical sociodemographic differences in coping strategies. Additionally, most existing studies on climate anxiety have been conducted in high-income countries; this study provides a critical perspective from a low-middle-income country, where children are disproportionately exposed to the physical and emotional consequences of climate crises. Our findings underscore the urgency of integrating mental health services into climate adaptation strategies, particularly in under-resourced contexts. This is especially relevant for Generation Z and Generation Alpha, who are growing up amid escalating climate uncertainty. Recent work by Sanson et al. [34] and Burke et al. [35] shows that climate anxiety among these generations is often accompanied by diminished trust in adults and governments, which can further compound feelings of helplessness and distress. The data from Maiduguri, Nigeria, thus provides a unique lens into the intersection of climate anxiety, armed conflict, and socioeconomic vulnerability with implications for specific mental health interventions.

While the SDQ-13 used in this study was not designed as a diagnostic tool aligned with DSM (Diagnostic and Statistical Manual)-5 criteria, its use in this study has been valuable in identifying children who exhibit high levels of psychological distress. The SDQ-13 captures emotional and behavioral difficulties that may signal underlying mental health concerns, such as anxiety and emotional withdrawal, which are consistent with symptoms of disorders like PTSD. However, the SDQ-13 alone cannot confirm a formal diagnosis, as it does not assess symptom duration or severity in the way DSM-5 criteria require for conditions like generalized anxiety disorder or PTSD. The DSM-5 provides a more nuanced framework for diagnosis, accounting for factors such as symptom frequency, duration, and functional impairment. Therefore, while the SDQ-13 helps screen children at risk for mental health issues [36], it should be supplemented with other diagnostic tools and clinical judgment to achieve a comprehensive assessment. Given the high levels of distress observed in this study, these findings should be viewed as an indication of potential psychological challenges, underscoring the need for early intervention. Early identification through such screenings can allow for proactive support [36], addressing emotional and behavioral difficulties before they escalate into diagnosable mental health disorders. Thus, while the SDQ-13 serves as an important screening tool, its results should be interpreted in conjunction with professional evaluation to inform appropriate mental health interventions.

### Limitations of the Study

Despite the strengths of this study, several limitations need to be considered while interpreting the findings. First, the study was conducted in Maiduguri, a region exposed to compounded and chronic stressors, including prolonged armed conflict, large-scale displacement, and seasonal flooding. These overlapping adversities complicate the ability to isolate the psychological effects of flooding alone. While the 2024 flood was the most recent and prominent disaster, it occurred in a context of longstanding trauma, and children’s psychological responses may reflect cumulative exposure rather than the flood event alone. Notably, the severity of the 2024 flood was unprecedented due to the collapse of the city’s main water supply dam, intensifying destruction and displacement. Second, data were collected through parental reports rather than directly from the children. Although this method was ethically necessary to minimize potential harm and ensure informed consent, it may have reduced the reliability of the information provided, as parents might not fully capture the nuanced emotional states or coping behaviors of their children. Third, of the 384 questionnaires distributed, only 374 met the criteria for analysis, resulting in a slightly smaller sample than intended, which may limit the generalizability of the findings. Fourth, the use of a cross-sectional design restricts causal interpretations. The study identifies associations between psychological distress and coping strategies, but cannot establish cause-and-effect relationships. Fifth, we noted the limitation concerns the categorization of coping strategies into discrete levels (e.g., poor, moderate, strong). While this approach aids interpretability, it may introduce issues such as loss of statistical power, potential misclassification near threshold values, and reduced sensitivity to individual differences. Future research may benefit from analyzing coping scores as continuous variables or employing more sophisticated classification methods, such as latent profile analysis, to capture the complexity of coping behaviors more accurately. Sixth, while this study focused on the psychological impact of the 2024 flood, it is important to note that it did not assess specific trauma exposures such as child marriage or family separation, which are more directly linked to the longstanding insurgency and displacement in the region. Future research should integrate these factors to better capture the cumulative adversity affecting children’s mental health.

Finally, a significant limitation of this study is the absence of reported reliability testing for the scales used, specifically the Strengths and Difficulties Questionnaire (SDQ-13) and the KidCOPE parent version. Additionally, no specific cross-cultural validation was conducted for these instruments in the current study. While both tools have been widely used and culturally validated in similar low- and middle-income country settings, including those affected by disasters [14,16,37,38], their reliability and cultural appropriateness in this specific context remain untested. The SDQ-13 and KidCOPE parent version are both standardized instruments that have demonstrated reliability and validity in various studies, including those conducted in disaster-affected populations in sub-Saharan Africa and other comparable regions [14,16,37,38]. However, the lack of explicit reliability testing in the present study limits our ability to assess the internal consistency of these tools within the unique cultural and environmental conditions of Maiduguri, Nigeria. Future research should prioritize the inclusion of reliability statistics and cross-cultural validation to ensure that these instruments remain robust and culturally relevant in diverse contexts. The inclusion of such data would strengthen the findings and provide greater confidence in the use of these instruments for assessing psychological distress and coping mechanisms in disaster-affected populations.

## 5. Conclusions

This study found that a majority of flood-affected children in Maiduguri, Nigeria, experience significant psychological distress, with many children affected by the floods exhibiting maladaptive coping strategies and lacking robust adaptive coping mechanisms. This distress was influenced by several factors, including disrupted education, poverty, and ongoing trauma; however, no sociodemographic variable independently predicted coping styles after adjustment.

Based on the aforementioned findings, the current study has several implications. There is an urgent need to establish community-based mental health and psychosocial support services for children affected by floods. This should include structured coping skills training programs tailored to children’s developmental stages and trauma histories, particularly in low-resource and conflict-affected settings like Maiduguri. Improving access to formal education for displaced or out-of-school children is also critical, as schools can serve as key platforms for early identification and support. Moreover, equipping parents and caregivers through targeted psychoeducation and support interventions can enhance protective environments for children. These findings also underscore the need to integrate child-focused mental health care into emergency preparedness frameworks and disaster response strategies. For clinical practice, this calls for the development of culturally appropriate screening protocols and intervention models. For policy and training, health and education professionals should receive capacity building support on trauma-informed care and community-level mental health promotion. Lastly, intersectoral collaboration across health, education, child protection, and humanitarian sectors is vital to ensure a comprehensive, sustainable, and contextually relevant response to the psychological needs of vulnerable children.

## Figures and Tables

**Table 1:** Sociodemographic characteristics of respondents and study variables.

Variables	Frequency	Percentage (%)
Age	11.2 ± 3.1 (Mean ± SD)	100
Gender		
Male	210	56.1
Female	164	43.9
Level of education of the child		
Not in any formal education	154	41.2
Primary education	130	34.8
Secondary education	90	24.1
Level of education of the father		
No formal education	203	54.3
Primary	113	30.2
Secondary	35	9.4
Tertiary	23	6.1
Religious status		
Islam	236	63.1
Christianity	138	36.9
Household income per month (Naira)		
5000–20,000	116	31.0
21000–30000	155	41.4
31,000–50,000	51	13.6
>50,000	52	13.9
Psychological distress		
Normal	136	36.4
Abnormal	238	63.6
Maladaptive coping		
Low	139	37.2
Moderate	163	43.6
High	72	19.3
Adaptive coping		
Poor	201	53.8
Moderate	165	44.1
Strong	8	2.1

SD: Standard Deviation.

**Table 2:** Relationship between psychological distress with levels of coping mechanisms (maladaptive and adaptive).

Coping Level Variable	OR (95% CI) of Psychological Distress
Maladaptive coping	
Low (ref)	1
Moderate	1.72 *** (1.25–2.36)
High	2.43 *** (1.46–4.04)
Adaptive coping	
Poor (ref)	1
Moderate	1.90 *** (1.38–2.61)
Strong	1.00 (0.25–4.00)

OR: odds ratio; CI: confidence intervals; *** *p* < 0.001.

**Table 3:** Logistic regression predicting sociodemographic factors associated with psychological distress.

Variables	Crude OR (95% CI)	Adjusted OR (95% CI)
Age	1.05 *** (1.03–1.06)	1.00 (0.96–1.04)
Gender		
Male (ref)	1	1
Female	1.65 ** (1.20–2.26)	0.92 (0.60–1.40)
Child education		
No formal (ref)	1	1
Primary	1.89 *** (1.32–2.71)	1.26 (0.77–2.06)
Secondary	1.81 ** (1.18–2.79)	1.43 (0.82–2.48)
Father’s education		
No formal (ref)	1	1
Primary	2.42 *** (1.62–3.64)	1.53 (0.92–2.55)
Secondary	1.33 (0.68–2.60)	0.93 (0.44–1.95)
Tertiary	2.29 (0.94–5.56)	1.89 (0.72–4.95)
Religion		
Islam (ref)	1	1
Christianity	2.14 *** (1.49–3.06)	1.52 (0.97–2.38)
Household Income (Naira)		
5000–20,000 (ref)	1	1
21,000–30,000	1.98 *** (1.42–2.77)	1.36 (0.83–2.22)
31,000–50,000	1.04 (0.60–1.80)	0.76 (0.40–1.45)
>50,000	2.25 ** (1.25–4.05)	1.53 (0.77–3.07)

OR: odds ratio; CI: confidence intervals; ** *p* < 0.01, *** *p* < 0.001.

**Table 4:** Ordinal logistic regression predicting sociodemographic factors associated with adaptive and maladaptive coping.

Variables	Adaptive Estimate (95% CI)	Maladaptive Estimate (95% CI)
Age	0.030 (–0.04, 0.10)	0.02 (–0.05, 0.08)
Gender (Female = ref)		
Male	–0.09 (–0.50, 0.32)	–0.04 (–0.43, 0.35)
Child education (Tertiary = ref)		
Primary	0.14 (–0.40, 0.68)	–0.07 (–0.58, 0.44)
Secondary	0.27 (–0.29, 0.82)	–0.14 (–0.66, 0.39)
Father’s education (Tertiary = ref)		
No formal	0.49 (–0.42, 1.41)	0.38 (–0.47, 1.23)
Primary	0.31 (–0.66, 1.27)	0.27 (–0.62, 1.16)
Secondary	0.27 (–0.84, 1.38)	–0.22 (–1.25, 0.82)
Religion (Christianity = ref)		
Islam	–0.02 (–0.46, 0.43)	0.08 (–0.34, 0.50)
Economic status in Naira (>50,000 = ref)		
5000–20,000 (ref)	0.06 (–0.62, 0.73)	0.45 (–0.18, 1.08)
21,000–30,000	0.03 (–0.62, 0.68)	0.03 (–0.57, 0.64)
31,000–50,000	0.36 (–0.43, 1.14)	–0.40 (–1.15, 0.34)

CI: confidence intervals.

## Data Availability

The original contributions presented in this study are included in the article and Supplementary Materials. Further inquiries can be directed to the corresponding author.

## Supplementary Materials

The following supporting information can be downloaded at: https://www.mdpi.com/article/10.3390/children12091137/s1, Figure S1: Psychological distress by level of adaptive coping; Figure S2: Psychological distress by level of maladaptive coping.

## References

[1] WHO Regional Office for Africa (2024). Delivering Lifesaving Health Services for Flood-Displaced Families in Nigeria.

[2] Save the Children (2024). Nigeria Floods: Three Million Children in Borno State Without School and Exposed to Child Marriage, Malnutrition and Disease.

[3] Giovanelli A., Mondi C.F., Reynolds A.J., Ou S.-R. (2020). Adverse childhood experiences: Mechanisms of risk and resilience in a longitudinal urban cohort. Dev. Psychopathol..

[4] Michael T.O. (2024). Adapting to climate change-induced flooding: Insights from women traders in the riverine areas of Nigeria—A qualitative study. Front. Sustain..

[5] Mohammed Ibrahim A.B., Adeyemi A.I., Abubakar M. (2021). Modelling Flood Scenarios in Northeast Nigeria: Simulations, Vulnerabilities and Implications. Afr. J. Earth Environ. Sci..

[6] Abbas A., Ago H.A., Bukar M., Abare S.M., Hassan U. (2024). Impact of Displacement and Adjustment on Girl-Child Education in Mega Schools in Maiduguri Metropolis, Borno State, Nigeria. J. Contemp. Educ. Res..

[7] Adeleke O.A., Fadairo O.S., Tella A.M., Oyewole M.F. (2024). Coping Strategies Used by Flood Victims in Rural Households of Benue State, Nigeria. J. Agric. Ext..

[8] Ayuba H., Monguno A.K., Joseph W., Madueke K.L., Buba I., Bukar B., Abubakar A.A., Abubakar Y., Ajadi S.B. (2024). Lessons in Urban Resilience from the Floods in Maiduguri, Nigeria.

[9] Masten A.S., Narayan A.J. (2012). Child development in the context of disaster, war, and terrorism: Pathways of risk and resilience. Annu. Rev. Psychol..

[10] Alexander L., Lopes B., Ricchetti-Masterson K., Yeatts K. (2017). Case-control studies. Eric NoteBook.

[11] Setia M.S. (2016). Methodology series module 3: Cross-sectional studies. Indian J. Dermatol..

[12] Agbaje O.S., Nnaji C.P., Nwagu E.N., Iweama C.N., Umoke P.C.I., Ozoemena L.E., Abba C.C., Agbaje O.S., Nnaji C.P., Nwagu E.N. (2021). Adverse childhood experiences and psychological distress among higher education students in Southeast Nigeria: An institutional-based cross-sectional study. Arch. Public Health.

[13] Kar N. (2024). Coping strategies used by children and adolescents following disaster trauma: A review of associated factors and intervention options. Odisha J. Psychiatry.

[14] Cherewick M., Bertomen S., Njau P.F., Leiferman J.A., Dahl R.E. (2024). Dimensions of the KidCope and their associations with mental health outcomes in Tanzanian adolescent orphans. Health Psychol. Behav. Med..

[15] Daniel W.W., Cross C.L. (2018). Biostatistics: A Foundation for Analysis in the Health Sciences.

[16] Brown A., Weiss E., Suntheimer N.M., Appiah R., Aurino E., Wolf S. (2025). Factorial and cultural validity of a social and emotional behavior measure in Northern Ghana. Appl. Dev. Sci..

[17] World Medical Association Declaration of Helsinki: Ethical Principles for Medical Research Involving Human Subjects. https://www.wma.net/policies-post/wma-declaration-of-helsinki-ethical-principles-for-medical-research-involving-human-subjects/.

[18] Masiran R., Tan K.-A., Ismanizan M.A., Roslee N.A., Prabaharan P. (2025). Prevalence of children’s emotional and behavioural problems and parents’ psychological distress in child and adolescent psychiatric clinic Hospital Sultan Abdul Aziz Shah. Int. J. Psychiatry Clin. Pract..

[19] Hickman C., Marks E., Pihkala P., Clayton S., Lewandowski R.E., Mayall E.E., Wray B., Mellor C., Van Susteren L. (2021). Climate anxiety in children and young people and their beliefs about government responses to climate change: A global survey. Lancet Planet. Health.

[20] David O.P., Dammeyer J., Dangana J.M. (2023). Experiences of mental health problems vulnerability, psychological symptoms and coping mechanisms of displaced adolescents in North-east Nigeria. Afr. Health Sci..

[21] Cénat J.M., Farahi S.M.M.M., Rousseau C., Bukaka J., Darius W.P., Derivois D., Dalexis R.D., Luyeye N. (2023). Prevalence and factors related to post-traumatic stress disorder and depression symptoms among children and adolescents survivors and orphans of Ebola virus disease in Democratic Republic of the Congo eastern regions during the COVID-19 pandemic. J. Adolesc. Health.

[22] Ghasemi F., Beversdorf D.Q., Herman K.C. (2024). Stress and stress responses: A narrative literature review from physiological mechanisms to intervention approaches. J. Pac. Rim Psychol..

[23] Wadsworth M.E. (2015). Development of maladaptive coping: A functional adaptation to chronic, uncontrollable stress. Child Dev. Perspect..

[24] Sheikh T.L., Mohammed A., Agunbiade S., Ike J., Ebiti W.N., Adekeye O. (2014). Psycho-trauma, psychosocial adjustment, and symptomatic post-traumatic stress disorder among internally displaced persons in Kaduna, Northwestern Nigeria. Front. Psychiatry.

[25] Raccanello D., Rocca E., Barnaba V., Vicentini G., Hall R., Brondino M. (2023). Coping strategies and psychological maladjustment/adjustment: A meta-analytic approach with children and adolescents exposed to natural disasters. Child Youth Care Forum.

[26] Pfefferbaum B., Jacobs A.K., Griffin N., Houston J.B. (2015). Children’s disaster reactions: The influence of exposure and personal characteristics. Curr. Psychiatry Rep..

[27] Majumder J., Saha I., Bagepally B.S., Kalita M., Munikrishnappa D., Ray S., Saha A., Chakrabarti A. (2024). Mental health burden following extreme weather events in South-east Asia: A systematic review and meta-analysis. Indian J. Psychiatry.

[28] Gebremedhin H.T., Bifftu B.B., Lebessa M.T., Weldeyes A.Z., Gebru T.T., Petrucka P. (2020). Prevalence and associated factors of psychological distress among secondary school students in Mekelle city, Tigray region, Ethiopia: Cross-sectional study. Psychol. Res. Behav. Manag..

[29] Tol W.A., Le P.D., Harrison S.L., Galappatti A., Annan J., Baingana F.K., Betancourt T.S., Bizouerne C., Eaton J., Engels M. (2023). Mental health and psychosocial support in humanitarian settings: Research priorities for 2021–30. Lancet Glob. Health.

[30] Đurišić M., Bunijevac M. (2017). Parental involvement as a important factor for successful education. Cent. Educ. Policy Stud. J..

[31] Lefevor G.T., Etengoff C., Davis E.B., Skidmore S.J., Rodriguez E.M., McGraw J.S., Rostosky S.S. (2023). Religion/spirituality, stress, and resilience among sexual and gender minorities: The religious/spiritual stress and resilience model. Perspect. Psychol. Sci..

[32] Sinyor M., Silverman M., Pirkis J., Hawton K. (2024). The effect of economic downturn, financial hardship, unemployment, and relevant government responses on suicide. Lancet Public Health.

[33] Batte C., Nuwasiima S., Semulimi A.W., Apio P.O., Mutebi R.K., Mwesigwa M.M., Twinamasiko N., Siddharthan T., Mukisa J., Mukunya D. (2024). Coping strategies of school-going adolescents during the COVID-19 pandemic in the climate vulnerable Manafwa watershed, Uganda. BMC Psychol..

[34] Sanson A.V., Van Hoorn J., Burke S.E. (2019). Responding to the impacts of the climate crisis on children and youth. Child Dev. Perspect..

[35] Burke S.E., Sanson A.V., Van Hoorn J. (2018). The psychological effects of climate change on children. Curr. Psychiatry Rep..

[36] Goodman R., Ford T., Simmons H., Gatward R., Meltzer H. (2000). Using the Strengths and Difficulties Questionnaire (SDQ) to screen for child psychiatric disorders in a community sample. Br. J. Psychiatry.

[37] Polanczyk G.V., Salum G.A., Sugaya L.S., Caye A., Rohde L.A. (2015). Annual research review: A meta-analysis of the worldwide prevalence of mental disorders in children and adolescents. J. Child Psychol. Psychiatry.

[38] Cartwright K., El-Khani A., Subryan A., Calam R. (2015). Establishing the feasibility of assessing the mental health of children displaced by the Syrian conflict. Glob. Ment. Health.

