# Supplementary material for: Psychological Distress and Coping Mechanisms Among Flood-Affected Children in Maiduguri, Nigeria
Source: Children (Basel). 2025 Aug 28;12(9):1137. doi: 10.3390/children12091137 (PMC12468849; doi:10.3390/children12091137)

Figure S1: Psychological distress by level of adaptive coping

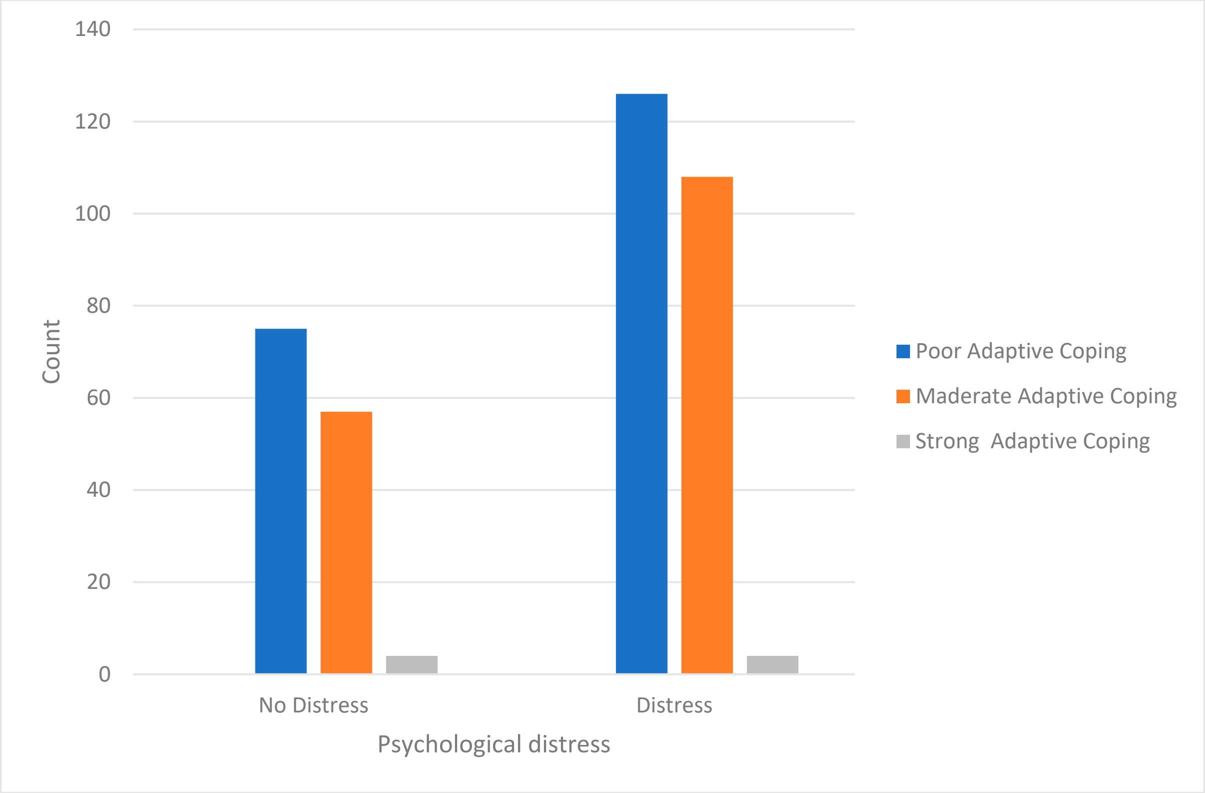


Figure S2:  Psychological distress by level of maladaptive coping

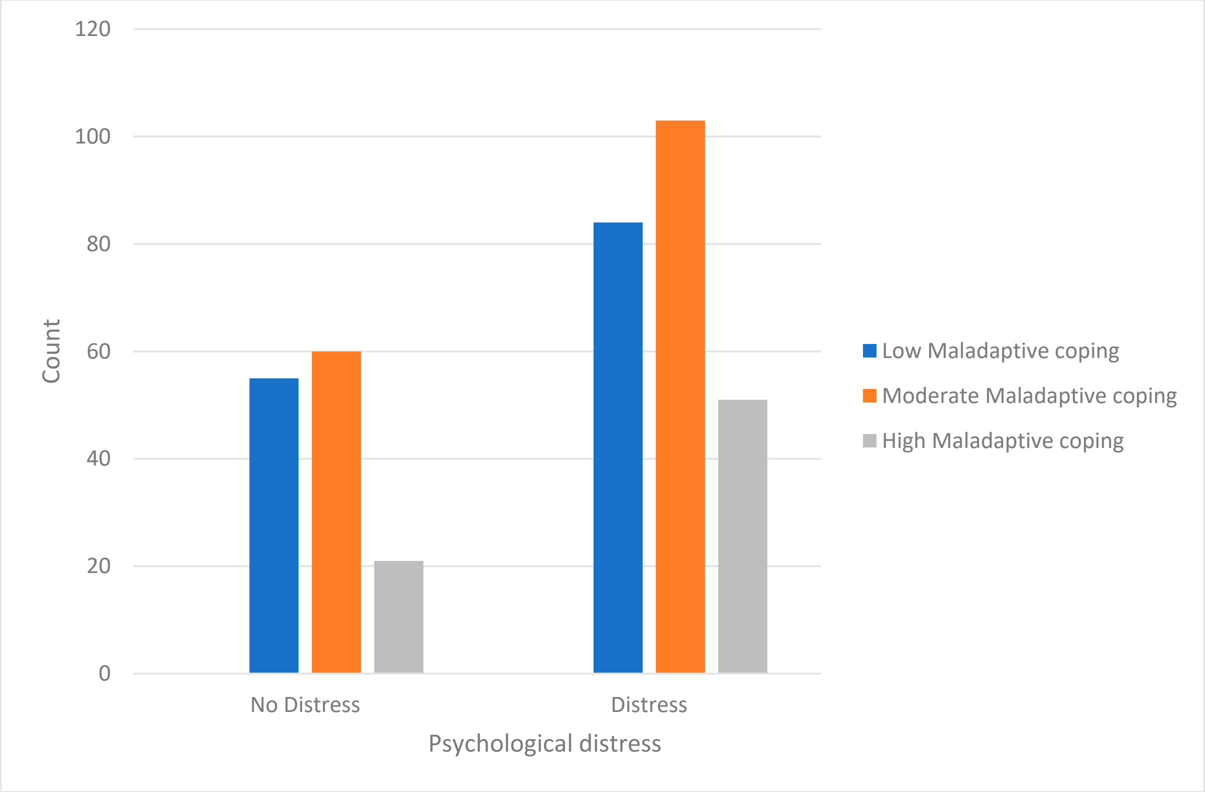

Supplement: Supplementary file 1 [file children-12-01137-s001.zip › children-3741130-supplementary.pdf]
